# Effects of human trampling on abundance and diversity of vascular plants, bryophytes and lichens in alpine heath vegetation, Northern Sweden

**DOI:** 10.1186/s40064-015-0876-z

**Published:** 2015-02-26

**Authors:** Annika K Jägerbrand, Juha M Alatalo

**Affiliations:** VTI, Swedish National Road and Transport Research Institute, Box 55685, 102 15 Stockholm, Sweden; Department of Ecology and Genetics, Uppsala University, Campus Gotland, SE-621 67 Visby, Sweden

**Keywords:** Cover, Disturbance, Tundra, Richness, Evenness, Trail, Hiking

## Abstract

This study investigated the effects of human trampling on cover, diversity and species richness in an alpine heath ecosystem in northern Sweden. We tested the hypothesis that proximity to trails decreases plant cover, diversity and species richness of the canopy and the understory. We found a significant decrease in plant cover with proximity to the trail for the understory, but not for the canopy level, and significant decreases in the abundance of deciduous shrubs in the canopy layer and lichens in the understory. Proximity also had a significant negative impact on species richness of lichens. However, there were no significant changes in species richness, diversity or evenness of distribution in the canopy or understory with proximity to the trail. While not significant, liverworts, acrocarpous and pleurocarpous bryophytes tended to have contrasting abundance patterns with differing proximity to the trail, indicating that trampling may cause shifts in dominance hierarchies of different groups of bryophytes. Due to the decrease in understory cover, the abundance of litter, rock and soil increased with proximity to the trail. These results demonstrate that low-frequency human trampling in alpine heaths over long periods can have major negative impacts on lichen abundance and species richness. To our knowledge, this is the first study to demonstrate that trampling can decrease species richness of lichens. It emphasises the importance of including species-level data on non-vascular plants when conducting studies in alpine or tundra ecosystems, since they often make up the majority of species and play a significant role in ecosystem functioning and response in many of these extreme environments.

## Background

Human recreational activities cause mechanical disturbances in natural ecosystems with undesirable effects on vegetation, such as changes in cover, species composition, diversity, plant height and increased risk of invasive species or weeds (Scott and Kirkpatrick 1994; Cole 2004; Pickering and Growcock 2009; Crisfield et al. 2012; Barros et al. 2013). Other effects of recreational activities include soil compaction impact, reducing the water-holding capacity of soils, which in turn may increase erosion rates and water run-off. Tourism in natural and wilderness ecosystems is popular and increasing worldwide (Buckley 2000). Unfortunately, even very low levels of visitor traffic can cause ecological changes (Forbes et al. 2004; Pertierra et al. 2013), and the wear and tear of human trampling and other activities reduces the environmental value for recreation (Forbes et al. 2004).

Most previous studies of human disturbance of vegetation have focused on the impacts on vascular plants (Cole 1995b; Whinam and Chilcott 1999; Cole and Monz 2002; Whinam and Chilcott 2003; Pickering and Growcock 2009; Bernhardt-Römermann et al. 2011; Barros et al. 2013; Pescott and Stewart 2014), while the impacts on plant community composition, bryophytes or lichens are less well documented (cf. Gremmen et al. 2003; Crisfield et al. 2012; Pertierra et al. 2013). Polar, alpine and tundra ecosystems are generally considered to be sensitive and fragile to disturbance and slow to recover, due to, for example low productivity, short growing season and a harsh climate, in combination with poor soil conditions. For example, in maritime Antarctica, each pedestrian transit creates a direct impact on fellfield vegetation (Pertierra et al. 2013). In addition, in Rocky Mountain national park, USA, the regeneration of severely degraded alpine tundra after trampling will probably take more than a century (Willard et al. 2007).

Bryophytes and lichens at high latitudes play a significant role in terms of species richness (Longton 1982; Matveyeva and Chernov 2000), biomass (Longton 1984; Nash 1996; Lange et al. 1998) and nutrient cycling (Crittenden 1983; Longton 1984; Nash 1996; Kielland 1997; Longton 1997). Furthermore, towards higher latitudes, the relative abundance of bryophytes and lichens increases as an indirect effect of the more rapid decline in vascular plant species richness (Vitt and Pakarinen 1977; Wielgolaski et al. 1981). Consequently, bryophytes and lichens are significant parts of ecosystem functioning in areas that are considered to be particularly vulnerable to human disturbance.

Studies on trampling impacts in arctic, antarctic and alpine tundra show that vascular plant species richness and diversity can sometimes decrease on and next to trails (Gremmen et al. 2003; Crisfield et al. 2012), but not always (Monz 2002; Hill and Pickering 2006; Crisfield et al. 2012). Trampling effects on the species richness and diversity of bryophytes and lichens have seldom been examined (cf. Gremmen et al. 2003), and the impacts on abundance and cover vary. Abundance of both bryophytes and lichens may be reduced (Grabherr 1982; Monz 2002; Pertierra et al. 2013) or increased (Törn et al. 2006; Gremmen et al. 2003), and there may be a delayed reduction in bryophyte abundance (Törn et al. 2006). Thus, considering the importance of bryophytes and lichens in alpine and tundra ecosystems, more studies on the effects of trampling are needed, particularly studies that include the impact on the whole community level, on species diversity, species richness and abundance of vascular plants, bryophytes and lichens. This study investigated trampling effects on species abundance, species richness and diversity at whole community level in a high alpine heath ecosystem in northern Sweden by analysing plant, bryophyte and lichen composition in transects perpendicular to a permanent trail used at low frequency by hikers.

We cannot assume that the ecosystem in this study will have any specific responses to trampling since previous studies show rather arbitrary or complex effects from trampling on vascular plants and there are few studies on bryophytes and lichen. However, since alpine heath ecosystem might be vulnerable to trampling in general, but in particular those who have a high proportion of bryophytes and lichens that are sensitive to trampling, it would seem likely to assume that proximity to trail could induce decreases of the vegetative cover, species richness and diversity of the canopy and understory.

Consequently, the following questions were asked: I) Does proximity to the trail cause a decrease in dominance of vascular plants, bryophytes and/or lichens? and II) Does species richness or diversity of vascular plants, bryophytes and/or lichens decrease with proximity to the trail?

## Results

**Figure 1:**
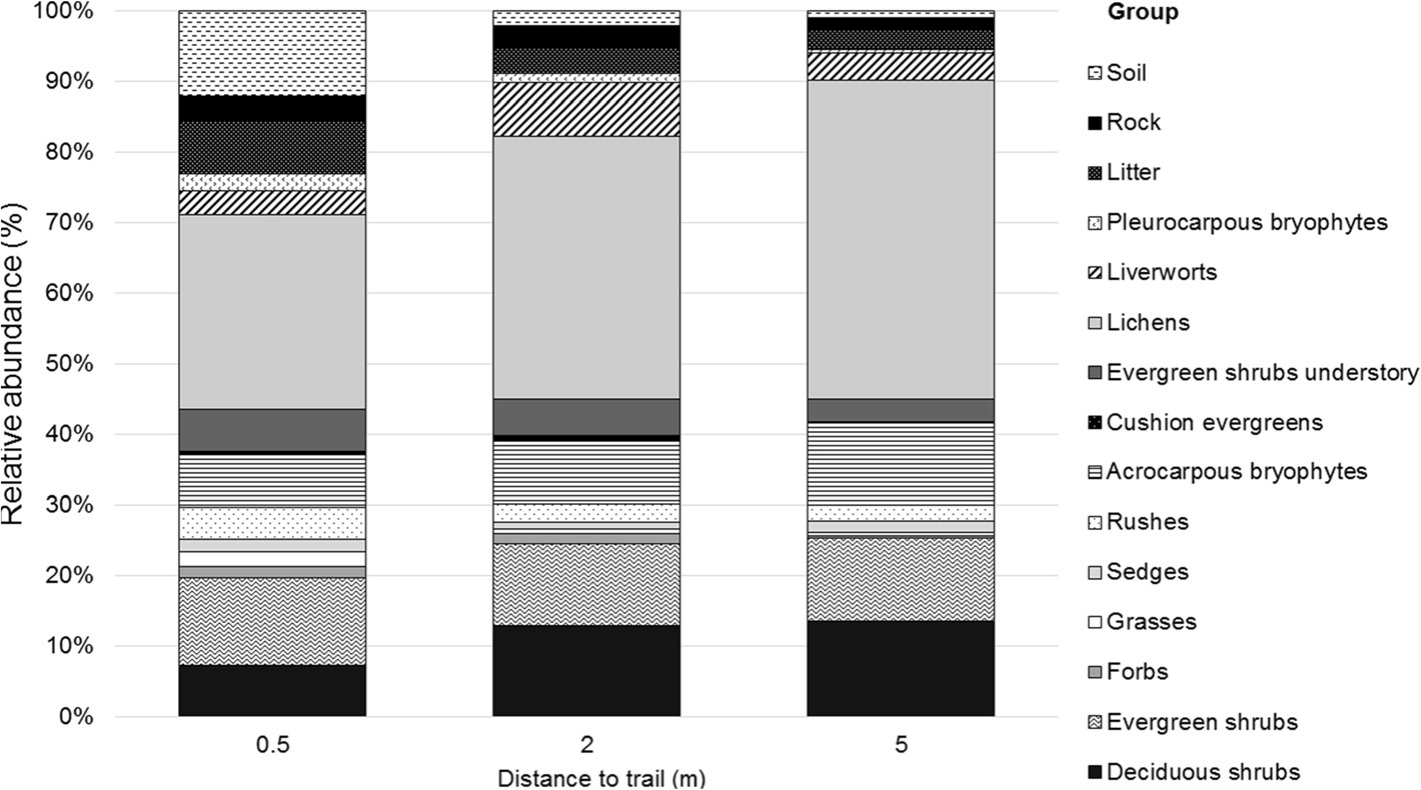
**Relative abundance (%) of different plants groups at 0.5, 2 and 5 m from the hiking trail in the Latnjavagge valley, northern Sweden.** Plants intercepted in the canopy layer were: deciduous shrubs, evergreen shrubs, forbs, grasses, sedges and rushes. Groups intercepted in the understory were: acrocarpous bryophytes, cushion evergreens, evergreen shrubs, lichens, liverworts, pleurocarpous bryophytes, litter, rock and soil, n =10.

A total of 26 vascular plant species were found in the canopy layer and three in the understory, while approximately 42 species of bryophytes and lichens were found in the understory (Table 1). The relatively undisturbed vegetation (5 m from the trail) was dominated by 28% shrubs (evergreen shrubs 15%, deciduous shrubs 14%) and 61% bryophytes and lichens, whereas the vegetation closest to the trail (within 0.5 m) was dominated by 26% shrubs (evergreen shrubs 18% and deciduous shrubs 7%) and 41% bryophytes and lichens. Thus there was a 20% decrease in bryophytes and lichens (Figure 1).

**Table 1:** **Species and groups divided into canopy and understory, at the Latnjavagge valley, northern Sweden**

**Canopy layer species**	**Group**
*Agrostis mertensii* Trin.	Grass
*Antennaria alpina* (L.) Gaertn.	Forb
*Betula nana* L.	Deciduous shrub
*Calamagrostis lapponica* (Wahlenb.) Hartm.	Grass
*Cassiope tetragona* (L.) D. Don.	Evergreen shrub
*Carex bigelowii* Torr. ex Schwein.	Sedge
*Empetrum nigrum* L.	Evergreen shrub
*Equisetum arvense* L.	Forb
*Erigeron uniflorus* L.	Forb
*Festuca ovina* L.	Grass
*Festuca vivipara* (L.) Sm.	Grass
*Hieracium* L. sect. Alpina (Griseb.) Gremli	Forb
*Juncus trifidus* L.	Rush
*Luzula arcuata* (Wahlenb.) Sw.	Rush
*Luzula spicata* (L.) DC.	Rush
*Minuartia biflora* (L.) Schinz & Thell.	Forb
*Poa alpina* L.	Grass
*Phyllodoce caerulea* (L.) Bab.	Evergreen shrub
*Pedicularis lapponica* L.	Forb
*Bistorta vivipara* (L.) Gray	Forb
*Salix herbacea* L.	Deciduous shrub
*Sibbaldia procumbens* L.	Forb
*Trisetum spicatum* (L.) K. Richter	Grass
*Taraxacum* sect. Taraxacum	Forb
*Vaccinium uliginosum* L.	Deciduous shrub
*Vaccinium vitis-idaea* L.	Evergreen shrub
**Understory species**	**Group**
*Alectoria nigricans* (Ach.) Nyl.	Lichen
*Alectoria ochroleuca* (Hoffm.) A. Massal.	Lichen
*Aulacomnium turgidum* (Wahlenb.) Schwägr.	Acrocarp
*Cassiope hypnoides* (L.) D. Don	Evergreen dwarf shrub
*Cetraria* sp.	Lichen
*Flavocetraria cucullata* (Bellardi) Kärnefelt & A. Thell	Lichen
*Cetrariella delisei* (Bory ex Schaer.) Kärnfelt & A. Thell	Lichen
*Cetraria islandica* (L.) Ach.	Lichen
*Flavocetraria nivalis* (L.) Kärnefelt & A. Thell	Lichen
*Cladonia arbuscula* (Wallr.) Flot.	Lichen
*Cladonia* sp.	Lichen
*Cladonia gracilis* (L.) Willd.	Lichen
*Cladonia uncialis* (L.) F. H. Wigg.	Lichen
*Diapensia lapponica* L.	Cushion evergreen
*Dicranum elongatum* Schleich. ex Schwägr.	Acrocarp
*Dicranum groenlandicum* Brid.	Acrocarp
*Dicranella subulata* (Hedw.) Schimp.	Acrocarp
*Gymnomitrion* sp.	Liverwort
*Hylocomium splendens* (Hedw.) Schimp.	Pleurocarp
*Icmadophila ericetorum* (L.) Zahlbr.	Lichen
*Kiaeria starkei* (F. Weber & D. Mohr) I. Hagen	Acrocarp
*Loiseleuria procumbens* (L.) Desv.	Evergreen shrub
*Lophozia lycopodioides* (Wallr.) Cogn.	Liverwort
*Nephroma arcticum* (L.) Torss.	Lichen
*Pertusaria dactylina* (Ach.) Nyl.	Lichen
*Peltigera aphthosa* (L.) Willd.	Lichen
*Peltigera scabrosa* Th. Fr.	Lichen
*Pleurocladula albescens* (Hook.) Grolle	Liverwort
*Pleurozium schreberi* (Willd. ex Brid.) Mitt.	Pleurocarp
*Polytrichum alpinum* Hedw.	Acrocarp
*Polytrichum juniperinum* Hedw.	Acrocarp
*Polytrichum piliferum* Hedw.	Acrocarp
*Polytrichum sexangulare* Floerke ex Brid.	Acrocarp
*Pohlia nutans* (Hedw.) Lindb.	Acrocarp
*Psoroma hypnorum* (Vahl) Gray	Lichen
*Ptilidium ciliare* (L.) Hampe	Liverwort
*Racomitrium lanuginosum* (Hedw.) Brid.	Acrocarp
*Solorina crocea* (L.) Ach.	Lichen
*Sphaerophorus globosus* (Huds.) Vain.	Lichen
*Stereocaulon alpinum* Laurer	Lichen
*Stereocaulon* sp.	Lichen
*Sanionia uncinata* (Hedw.) Loeske	Pleurocarp
*Tetraplodon mnioides* (Hedw.) Bruch & Schimp.	Acrocarp
*Thamnolia vermicularis* (Sw.) Schaer.	Lichen
*Ochrolechia frigida* (Sw.) Lynge	Lichen

**Figure 2:**
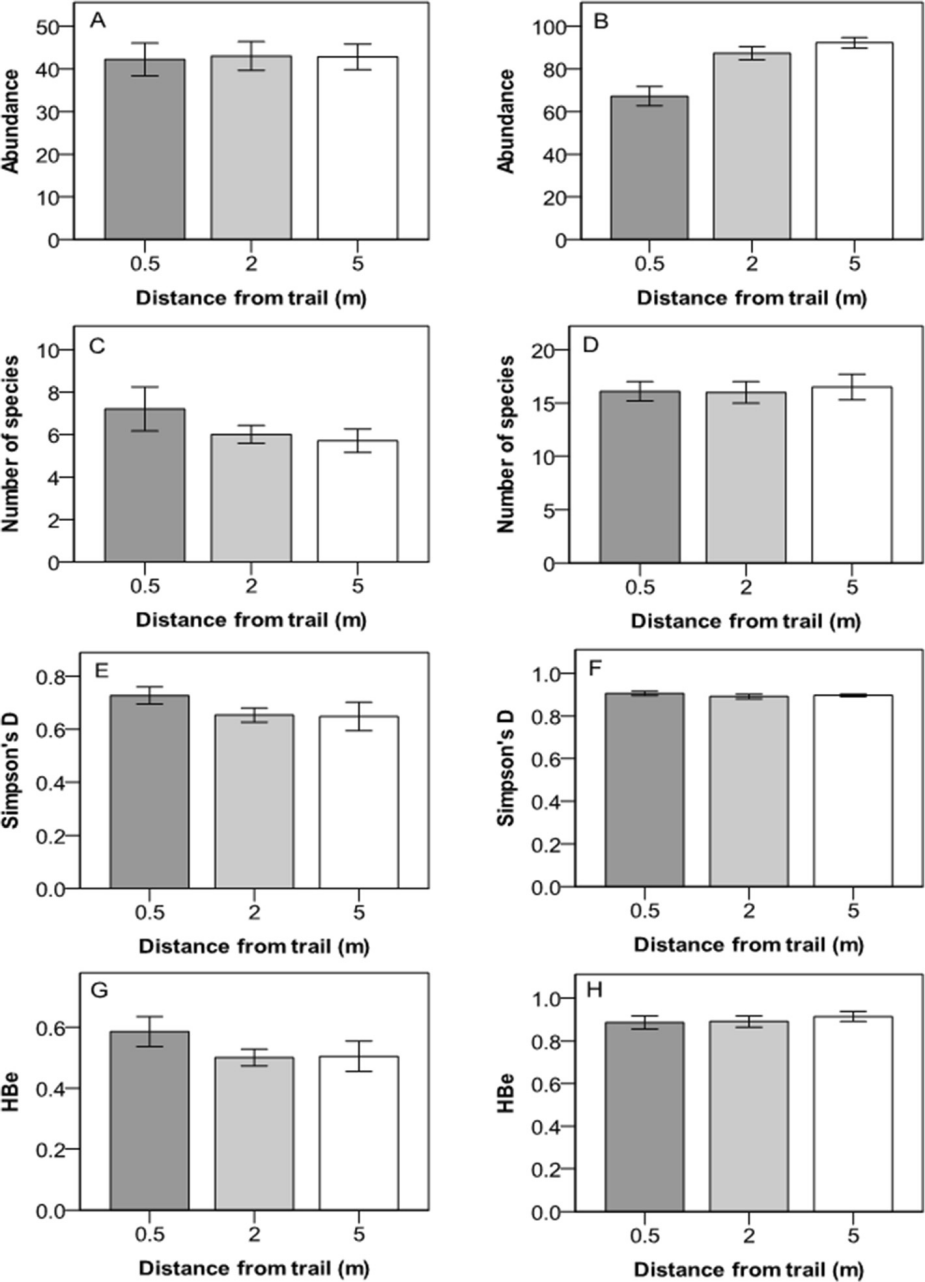
**Abundance, number of species, Simpson’s diversity index (D) and Brillouin evenness (HBe) for species in the canopy and understory at three different distances from the hiking trail in the Latnjavagge valley, northern Sweden.** Abundance in the **A)** canopy layer and **B)** understory, number of species in the **C)** canopy layer, and **D)** in the understory, Simpson’s diversity index in the **E)** canopy layer, and **F)** understory, Brillouin evenness in the **G)** canopy layer and **H)** understory. For species and plant functional groups, see Table 1. Mean value ± 1 S.E; n =10.

Vegetative cover in the understory (but not in the canopy layer) decreased significantly with proximity to the trail (within 0.5 m and within 2 m). There were no significant differences with distance from the trail in terms of number of species, Simpson’s diversity index D or Brillouin’s evenness (HBe) of the canopy or understory (Figure 2, Table 2). The number of species in the understory showed a significant relationship with the direction of the transect (Table 2).

**Table 2:** **Significant results from generalised linear mixed models (GLMM) explaining effects on abundance (count number per plot), and number of species at different distances from the hiking trail and at different directions in the Latnjavagge valley, northern Sweden**

**Variable**	**Group**	**Intercept**		**Distance from trail (m)**				**Direction**	
				**0.5**		**2**		**East**	
		**Coef**	***P***	**Coef**	***P***	**Coef**	***P***	**Coef**	***P***
Abundance	Understory	0.7	<0.0001	-0.5	<0.0001	-0.9	0.033	0.09	0.43
	Deciduous shrubs	18.5	<0.0001	-9.1	0.009	-0.9	0.78	2	0.58
	Acrocarpous bryophytes	16.72	<0.0001	-5.9	0.08	-3.6	0.27	-0.6	0.84
	Lichens	60.3	<0.0001	-25.3	<0.001	-11.1	0.008	10.5	0.14
Number of species	Understory	15.08	<0.0001	-0.4	0.69	-0.5	0.62	3.6	0.019
	Lichens	8.7	<0.0001	-1.5	0.008	-1.1	0.045	3	0.054

Coef = coefficient, P = significance level, n =10.

**Figure 3:**
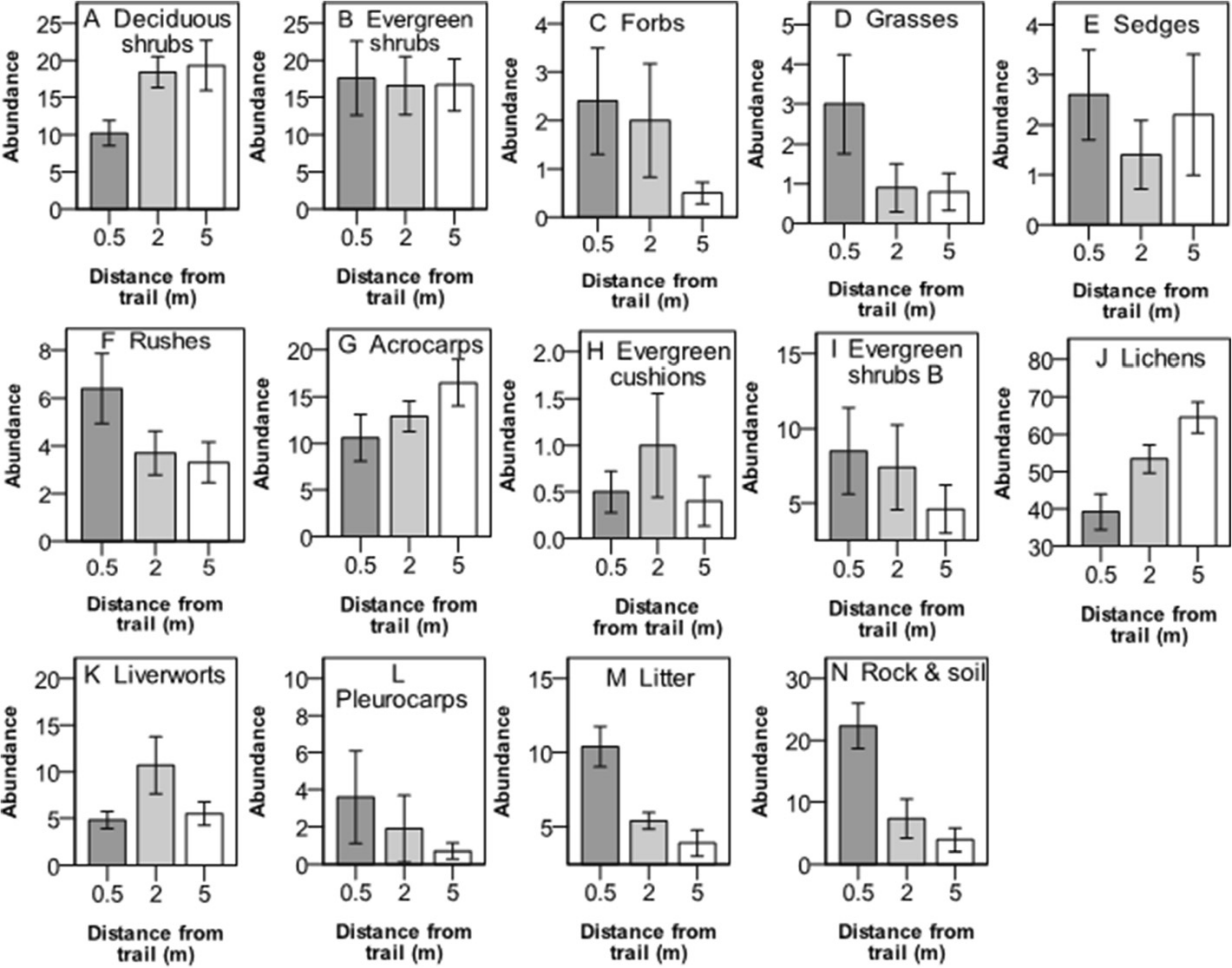
**Abundance (mean value ± 1 S.E.) of plant functional groups at three different distances from the hiking trail in the Latnjavagge valley, northern Sweden. A-F** show plant groups intercepted in the canopy layer and **G-N** show groups intercepted in the understory. For species and plant functional groups, see Table 1, n =10.

The abundance of deciduous shrubs was significantly lower closest to the trail (0.5 m), and lichens had significantly lower abundances at both 0.5 m and 2 m distance from the trail (Figure 3, Table 2). The opposite relationship was found for litter, rock and soil, all of which increased in abundance at 0.5 m (as an effect of decreased vegetation cover in the understory) (Figure 3, Table 3). Abundances of other plant functional groups were not significantly affected by proximity to the trail.

**Table 3:** **Results of the Mann-Whitney U-test**

**Tested groups (m from trail)**		**Litter**		**Soil and rock**	
		**Z**	**P**	**Z**	**P**
0.5	2	-3.02	0.03	-2.73	0.006
0.5	5	-3.04	0.002	-3.42	0.001
2	5	-1.77	0.076	-1.3	0.19

**Figure 4:**
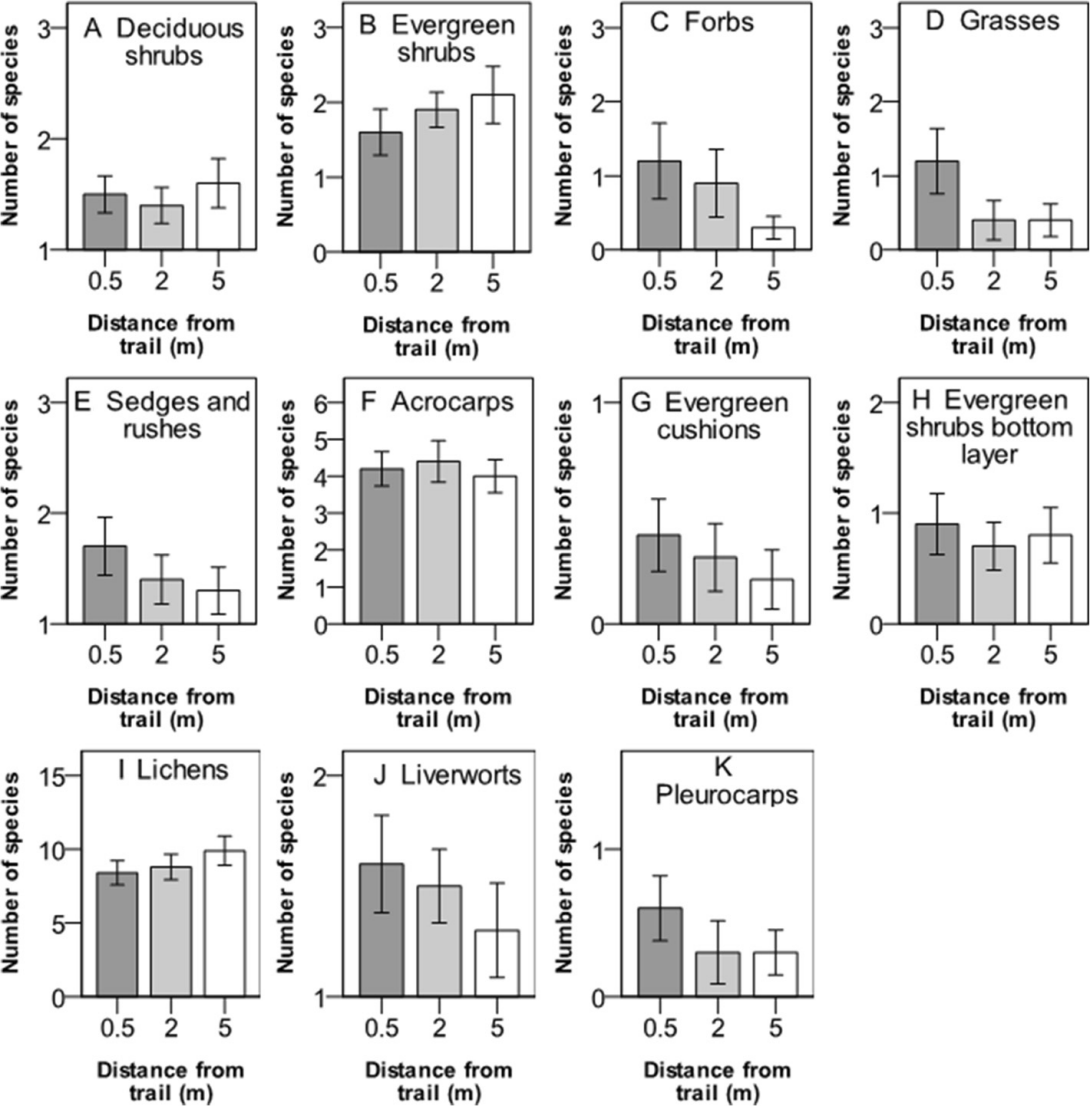
**Number of species (mean value ± 1 S.E.) of plant functional groups at three different distances from the hiking trail in the Latnjavagge valley, northern Sweden. A**-**E** show plant groups intercepted in the canopy layer and **F**-**K** show groups in the understory. For species and plant functional groups, see Table 1, n =10.

Proximity to the trail had a significant impact on the species richness of lichens, which decreased at 0.5 and 2 m from the trail, but no significant responses in species richness were found for the other functional plant groups studied (Figure 4, Table 2).

**Figure 5:**
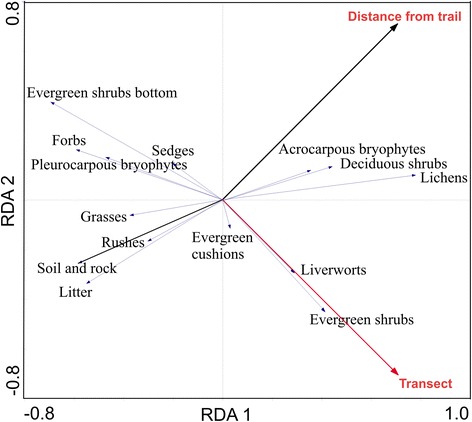
**Ordination diagram of redundancy analysis (RDA) showing the variation in groups, distance from trail (solid black arrow and red text) and transects (red arrow and red text) in the Latnjavagge valley, northern Sweden.** Groups in the canopy layer included deciduous shrubs, evergreen shrubs, forbs, grasses, sedges and rushes. Groups in the understory were: acrocarpous bryophytes, cushion evergreens, evergreen shrubs bottom, lichens, liverworts, pleurocarpous bryophytes, litter, rock and soil. For species and plant functional groups, see Table 1, n =10.

Multivariate analysis (RDA) explained 42% of the total variation, with RDA1 explaining 35% and RDA2 7% (Figure 5). Cumulative percentage variance of the groups-environment relationship was a total of 100% for the two first RDA axes and the RDA showed that distance to trail and the transect both significantly explained this variance (P < 0.001) and had similar model fit (0.21; F-ratio for distance 7.46, F-ratio for transect 9.75). Acrocarpous bryophytes, deciduous shrubs and lichens clustered together and were negatively affected by trampling, while litter, soil and rock, grasses, sedges and rushes seemed to be favoured by proximity to the trail (Figure 5). Clustering in other directions illustrates that the study area was somewhat heterogeneous in its species composition (Figure 5).

## Discussion

There was no significant decrease in vegetation cover at the canopy level with proximity to the trail, contradicting our initial hypothesis. However, proximity to the trail caused significant decreases in the abundance of deciduous shrubs in the canopy layer and total vegetative cover and lichens decreased with proximity to the trail in the understory, but no significant changes were found for the graminoids (grasses, sedges and rushes), forbs, bryophytes or plants in the understory. As a result of the decrease in vegetative cover of the understory, the abundance of litter, rock and soil increased with proximity to the trail. Reductions in cover or abundance of vascular plants have repeatedly been shown as a response to trampling effects and several studies show decreases in shrub cover (Cole 1995a; Whinam and Chilcott 1999; Gremmen et al. 2003; Whinam and Chilcott 2003; McDougall and Wright 2004; Barros et al. 2013; Ballantyne et al. 2014). Increases in litter, rock and soil abundance are also a well-established response to trampling (Cole 2004).

In general, graminoids as well as short, prostrate or rosette-forming plants are considered to be more tolerant to trampling than woody shrubs or taller forbs (e.g., Yorks et al. 1997; Bernhardt-Römermann et al. 2011; Ballantyne et al. 2014). In this study, there were no significant increases in abundance of evergreen shrubs or graminoids with proximity to the trail, but there was a tendency for this, especially for grasses and rushes, based on their positioning in the multivariate ordination diagram.

The significant reduction in vegetative cover in the understory with proximity to the trail was mainly driven by an significant decrease in lichens, from 45% at 5 m distance to 37% at 2 m from the trail and 28% at 0.5 m from the trail, resulting in a total reduction of 17%. There were no significant changes in total species richness, diversity or distribution in the canopy or understory with proximity to the trail, contradicting our initial hypothesis.

The results clearly show that trampling in alpine heaths has large negative impacts on lichen abundance and species richness. This is in line with previous observations that lichens are very trampling-sensitive in alpine grasslands (Grabherr 1982) and in maritime Antarctica (Pertierra et al. 2013), although to our knowledge this is the first study showing that trampling can decrease lichen species richness. However, experimental trampling in an alpine heath ecosystem in Scandinavia has shown the opposite effect. Instead of dramatic decreases in lichen abundance, as observed here, the trampling treatment increased the cover of lichens through regeneration (Törn et al. 2006). Although it is difficult to compare trampling with regeneration after the trampling pressure has been removed, the difference in impact may be due to differences in dominance prior to trampling. In the experimental trampling study (Törn et al. 2006), the lichen cover was only 8% and that of bryophytes was up to 50%, while at our study site the lichen cover was around 45% in the plots furthest away from the trail.

Our results from a lichen-dominated ecosystem in high alpine Sweden are therefore perhaps more directly comparable to trampling studies in maritime Antarctica, with a high coverage of bryophytes and/or lichens, than to other tundra ecosystems in, for example, Scandinavia, the Alps or America, with low amounts of non-vascular plants. As there are very few studies on species level of non-vascular plants, it is difficult to compare our results on the species richness of bryophytes and lichens with those of previous studies, where non-vascular plants are mostly treated as a group and data on species level are lacking. In the present study, species richness, diversity and evenness of distribution were always higher for the understory, which was dominated mainly by bryophytes and lichens, than the canopy layer, which was dominated by vascular plants. These results and those of others (Cornelissen et al. 2007; Turetsky et al. 2012) demonstrate the importance of including non-vascular plants when conducting studies in alpine or tundra ecosystems, when bryophytes and lichens often make up the majority of the species and play a significant role for ecosystem functioning.

The results also showed that bryophyte abundance as a group was not significantly related to proximity to the trail and that the three groups of bryophytes showed contrasting trends in abundance regarding proximity to the trail. While the differences were not significant, acrocarpous bryophytes tended to decrease with proximity to the trail, pleurocarpous bryophytes tended to increase with proximity to the trail and liverworts tended to peak at mid-range (2 m) distance from the trail. Thus, the results indicate that different levels of trampling may cause changes in dominance structures of the bryophyte communities over time. A potential explanation for the decline in acrocarpous species may be differences in physiology. Some bryophyte species and groups have structures for transporting water in their conducting tissues (Glime 2007), thereby giving them the capability for withstanding and tolerating trampling pressure in terms of impact on evapotranspiration or soil moisture conditions. Acrocarpous species have more advanced water-conducting structures, similar to “roots”, and may have been disadvantaged if proximity to the trail increased soil compaction, leading to a decrease in soil moisture. Pleurocarpous species may not have suffered this disadvantage, having more rudimental structures and lacking “roots”. In fact, soil compaction can lead to an increase in available surface water if it decreases the permeability of the soil.

Similarly, contrasting responses of bryophytes to trampling have been reported in the Subantarctic, where some species decreased in cover while others did not (Gremmen et al. 2003). Likewise, in maritime Antarctica, bryophyte communities dominated by different species had different tolerance to human trampling, but clearly better tolerance than the lichen-dominated fellfield (Pertierra et al. 2013). For example, acrocarpous mosses in mires and on slopes were more frequent at tracks than in controls, but the opposite was found for liverworts in mires (Gremmen et al. 2003). The reason that we found acrocarpous bryophytes to decrease with proximity to the track while Gremmen et al. (2003) found that they were more frequent in the tracks may be due to that fact that tracks in mires are more likely to be on drier land than the surrounding area in mires. Similar complex responses of different bryophytes to environmental change or experimental treatments have been shown previously in climate change experiments (Jägerbrand et al. 2009), but it is also reported that lichens and bryophytes exhibit considerable resistance to various warming simulations (Alatalo et al. 2014). Such responses might be explained by both bryophytes and lichens having somewhat different physiology to vascular plants since they can absorb water from the ambient air and are poikilohydric, i.e. able to suspend metabolism and thereby survive under dry conditions (Hosokawa et al. 1964; Proctor 1982; Proctor 1984; Proctor 1990).

It has been proposed that the changes in microclimate caused when vascular plant cover decreases as a trampling effect might have a greater impact on bryophytes in the longer term, through changes in light conditions and plant interactions, than direct trampling effects (Törn et al. 2006). In a previous study in a nearby alpine heath, the abundance of bryophytes was found to be negatively related to the abundance of shrubs and lichens (Jägerbrand et al. 2012). Thus, it seems difficult to separate bryophyte responses to changes in microclimatic conditions and those to negative interactions with other plants. Therefore, in order to fully understand the effect of human trampling on different bryophyte species or groups, it would be necessary to conduct factorial experimental manipulations. Future studies could make use of the presence of reindeers in the area and conduct comparative studies of human and mammal trampling. It would also be possible to conduct experiments comparing different usage levels of tourism similar to the study by Wolf and Croft (2014).

## Conclusions

Tourism in natural and wilderness ecosystems is popular and increasing worldwide (Buckley 2000). Unfortunately, even very low levels of visitor traffic can cause ecological changes (Forbes et al. 2004; Pertierra et al. 2013). This study investigated the effects of human trampling on cover, diversity and species richness in an alpine heath ecosystem in northern Sweden. We tested the hypothesis that proximity to trails decreases plant cover, diversity and species richness of the canopy and understory. We found a significant decrease in plant cover with proximity to the trail for the understory, but not for the canopy level, and significant decreases in the abundance of deciduous shrubs in the canopy layer and lichens in the understory. Proximity also had a significant negative impact on species richness of lichens. To our knowledge, this is the first study to demonstrate that trampling can decrease species richness of lichens. It emphasises the importance of including species-level data on non-vascular plants when conducting studies in alpine or tundra ecosystems, when they often make up the majority of species and play a significant role in ecosystem functioning and response in extreme environments.

## Materials and methods

### Study area

Fieldwork was conducted in northern Sweden, at the Latnjajaure Field Station, situated in the Latnjavagge valley (68°21′N, 18°29′E) at an elevation of 1000 m a.s.l. The climate at the site is characterised by cool summers and mild, snow-rich winters. The valley is completely to partially covered by snow for most of the year. Geographically and climatically, the valley is representative of the subarctic-alpine. However, based on the vegetation composition, the Latnjavagge area is more typically arctic (Molau and Alatalo 1998). The site has a mean annual temperature of -2.0 to -2.7°C (1993 to 1999) and mean annual precipitation of 808 mm (1990 to 1999), but the latter ranges from 605 mm (during 1996) to 990 mm (during 1993). The vegetation in the valley comprises a wide range of different species communities, since the area is geologically heterogeneous, ranging from acidic to basic rock, and varies from wet to dry (Jägerbrand et al. 2006).

This study was conducted in a dry poor heath community of the *Loiseleuria procumbens-Arctostaphylos alpinus-Empetrum hermafroditum*-type (Nordic Council of Ministers 1998) on acid glacial till. The study was performed at a site without permafrost on a flat terrain, below the southwest facing slope of Mt. Latnjatjårro. The area is homogenous regarding topography and soil conditions. The vascular plant richness is low and the vegetation is dominated by the shrubs *Betula nana* L., *Salix herbacea* L., *Empetrum nigrum* L., *Phyllodoce caerulea* (L.) Bab. and *Vaccinium vitis-idaea* L. and the rush *Juncus trifidus* L. The understory is species-rich, dominated by different bryophytes and lichens, for example, *Dicranum elongatum* Schleich. ex Schwägr., *Cladonia* species, *Stereocaulon alpinum* Laurer and *Ochrolechia frigida* (Sw.) Lynge.

There are no permanent inhabitants in the Latnjavagge valley. Latnjajaure Field Station has been used continuously as a field station during summer since 1990, but cannot be reached by vehicle during the snow-free period. The staff at Latnjajaure Field Station monitor the climate and long-term climate change experiments from late May or early June until early September. While there is no permanent tracking of the number of tourists passing through the valley, the number of tourists was noted by staff at the field station from 1999 to 2002. Between 1999-2002, there were approximately 319 (mean value, actual number varied between 281-399) hikers per year (Beylich et al. 2005). Visitors are much less common at other times of the year due to snow conditions. The trail in the valley is clearly marked and is made by people travelling by foot. Reindeer are occasionally found in the area, but have been observed to normally avoid the trail. The Abisko area (Abisko National Park) is a popular hiking destination that attracts approximately 50 000 visitors per year (Swedish Environmental Protection 2014). Most of these visit Kungsleden, a 440 km long hiking trail that is situated between Abisko in the north and Hemavan in the south. A side trail through the Latnjavagge valley has been in use for a long time (>50 years), but only a few hikers take this trail and, as observed by the staff at Latnjajaure Field Station, the number is fairly stable over the years (typically only a few people per day during the short summer period).

### Measurements

The effects of trampling on the plant community was investigated by randomly placing 10 transects perpendicular to the trail 5-20 meters apart and by using a 1 m × 1 m quadrat to point-frame three plots along each transect. Although the method of comparing trail-affected vegetation with adjacent undisturbed areas has some drawbacks (Cole 2004), we chose this method since we could not control the trampling in the area without extensive fencing, thus making experiments with before/after manipulations extremely difficult to implement. In fact, comparisons between disturbed and undisturbed vegetation may be the optimal way to study the long-term and repetitive impacts of hiking without causing additional impacts on the ecosystems with simulated trampling experiments, an important consideration in very sensitive ecosystems. This method of comparison has been used in many previous studies for analysing the impact of hiking and tourism (Grabherr 1982; Cole 2004; Crisfield et al. 2012).

The study area was chosen since it consisted of very homogeneous vegetation and was situated at a distance of approximately 300 m from the Latnjajaure Field Station, which was important to reduce trampling impacts by staff at the station. The distances between plots along the transect were chosen to represent three levels of hiking impact, based on visual determination of hiking impact. The first plot was placed at 0.5 m from the trail and had visibly fewer plants and more rocks and litter present. The two other plots represented low impact or undisturbed plots and were placed at 2 m and 5 m from the trail, in the surrounding vegetation. To enable sampling of 10 transects in a particular area of homogeneous vegetation composition, we placed transects 1,2,3,5,9 and 10 west of the trail and transects 4,6,7, and 8 in the east direction.

Species composition and abundance in the 30 plots were analysed with point-frame analysis (Walker 1996). This is a well-established measurement procedure for studying the abundance of plants in experiments simulating environmental change in tundra regions (Walker et al. 2006). We used a grid frame with 100 grid points spaced 10 cm apart to intercept 100 hits in two layers of the vegetation. The canopy layer was intercepted as the tallest plant hit in each of the 100 pins within the frame, and the understory, dominated by bryophytes, lichens and shorter vascular plants, was the layer intercepted beneath the canopy layer. Fieldwork took place between 29 July and 6 August 2013. The centre of the grid frame was placed exactly at the specific distances (0.5 m, 2 m and 5 m) from the trail.

A list of species intercepted in the canopy and understory, their names and plant functional groups can be found in Table 1. The abundance of functional types of plants, bryophytes and lichens was calculated as the sum of species tallies/hits per plot for each plant functional group. The following plant functional groups were used: deciduous shrubs, evergreen shrubs, forbs (i.e*.* herbaceous plants, including seedless vascular plants) grasses, sedges, rushes, bryophytes, lichens, evergreen cushions in the understory and evergreen shrubs in the understory (see e.g., Chapin et al. 1996; Arft et al. 1999). Bryophytes were divided into acrocarpous, pleurocarpous or liverworts based on their morphological and ecological differences. Nomenclature followed existing methods for vascular plants (Anderberg and Anderberg 2011), bryophytes (Swedish Museum of Natural History 2013a) and lichens (Swedish Museum of Natural History 2013b). For some taxa it was only possible to identify them to the level of genera (see Table 1).

### Data analyses

To test for trail impact, plots were compared at different distances from the trail. Abundance and number of species (species richness) were calculated per plot and separated into canopy and understory or into the different plant functional groups. Diversity was estimated by Simpson’s diversity index D (Simpson 1949) and the Brillouin-based evenness measure (HBe) was included as a measurement of the relative diversity and homogeneity of species (Pielou 1966). Prior to analyses, the data were tested for normality of distribution by the Shapiro-Wilks test (Shapiro and Wilks 1965). Variables meeting normality assumptions were subsequently analysed by generalised linear mixed models (GLMM), which test how one or more predictors can explain a target variable. We included the predictor variables distance from trail (0.5 m, 2 m, 5 m) and direction of transect (west or east) as fixed factors and transect number (1-10) as a random factor, and tested the significance of explaining the response variables. We used GLMM with an identity-link function assuming a linear scale response with maximum likelihood estimation. Response variables analysed by GLMM were: abundance of the canopy layer, number of species in the understory, Simpson’s diversity in the understory, Brillion’s evenness measure in the canopy and understory, abundance of deciduous shrubs, acrocarpous bryophytes, lichens, number of species of acrocarpous bryophytes, and lichens. Model fit was checked by viewing plots of predicted and observed residuals.

Variables not meeting the normality assumptions were analysed for response trends to the trail distance by the non-parametric Kruskal-Wallis test (Sokal and Rohlf 1995). When significant trends were revealed, the Mann-Whitney U-test was used to test for significant differences between trail distances (only used for the abundance of litter, rock and soil). The Shapiro-Wilks normality test, GLMM and non-parametric tests were all performed using IBM© SPSS© Statistics v. 19.0.02.

Community structure was analysed by detrended correspondence analysis (DCA), based on plant functional group abundances. DCA showed that the data were homogeneous and that the axes were of relatively short length, i.e. less than 2 standard deviation units for group turnover. This indicated that a linear multivariate technique would be more optimal for extracting the variation in the data. In order to investigate whether community structure was influenced by the variables trail distance, direction and transect number, we decided to use redundancy analysis (RDA), (see e.g., Ter Braak 1994), a multivariate gradient analysis that incorporates constrained factors to optimise the fit of the data based on linear assumptions. Significance of constraining factors was analysed by Monte Carlo permutation tests (1,000 permutations) on non-transformed data and default settings. DCA and RDA were performed in CANOCO 4.5 (Ter Braak and Šmilauer 2002).
